# Dual-Band CPW Graphene Antenna for Smart Cities and IoT Applications

**DOI:** 10.3390/s22155634

**Published:** 2022-07-28

**Authors:** Nathaniel Morales-Centla, Richard Torrealba-Melendez, Edna Iliana Tamariz-Flores, Mario López-López, Cesar Augusto Arriaga-Arriaga, Jesus M. Munoz-Pacheco, Victor R. Gonzalez-Diaz

**Affiliations:** 1 Faculty of Electronics Sciences, Autonomous University of Puebla, Puebla 72570, Mexico; nathaniel.morales@correo.buap.mx (N.M.-C.); mario.lopezlop@correo.buap.mx (M.L.-L.); cesarau.arriaga@correo.buap.mx (C.A.A.-A.); jesusm.pacheco@correo.buap.mx (J.M.M.-P.); vicrodolfo.gonzalez@correo.buap.mx (V.R.G.-D.); 2 Faculty of Computational Sciences, Autonomous University of Puebla, Puebla 72570, Mexico; iliana.tamariz@correo.buap.mx

**Keywords:** graphene, coplanar antenna, dual-band antenna, smart cities, internet of things

## Abstract

In this paper, a dual-band graphene coplanar waveguide antenna is designed for smart cities and internet of things applications. A graphene film is chosen as the conductive material for the radiation patches and ground plane with a thickness of 240 μm and an electric conductivity of 3.5 × 10^5^ S/m. The dielectric is glass with a dielectric permittivity of 6 and a thickness of 2 mm. The implementation of the antenna on glass permits the integration of the antenna in smart cities and IoT applications. This antenna is based on two trapezoidal patches that generate the dual-band behavior. The overall dimensions of the antenna are 30 mm × 30 mm × 2 mm. The reflection coefficient, gain, and radiation patterns were measured and compared with the simulations. The antenna covers two frequency bands; the lower band covers the 2.45 GHz ISM band, and the upper band range covers from 4 to 7 GHz.

## 1. Introduction

Nowadays, most electronic communications are wireless. Furthermore, the number of wireless links has increased in the last years due to the necessity to connect everything with everything [1]. Moreover, concepts such as the internet of things (IoT) [2,3,4], smart cities [5,6,7], and smart health [8,9], and vehicular networks such as vehicle to vehicle (V2V) or vehicle to infrastructure (V2I) [10,11,12] depend enormously on wireless sensor networks to perform. Antennas are a vital part of the wireless communications system in all these concepts. The antennas need to be placed in windows, buildings, clothing, vehicles, street infrastructure, and human bodies. These antennas must present a specific radiation and frequency operation performance. Moreover, the antennas have to be designed over novel materials that permit integration with the environment. This integration can be achieved using planar antennas fabricated over flexible and rigid materials; this material can also be opaque or transparent [13,14].

On the other hand, copper is usually the most employed in the conductor materials that conform to the antennas; however, recent research reports on using indium oxide (ITO) and graphene as a conductor in antenna design. Concerning ITO antennas, these present wideband behavior but low gain due to the low conductivity of the ITO, as reported in [15,16,17]. On the other hand, graphene presents properties such as high conductivity, flexibility, and excellent mechanical resistance [18,19]; this is ideal for it to be employed as a conductor in antennas. Due to these properties, graphene has been employed in antenna design for Terahertz communications [20,21,22] with a frequency range of 1 to 10 THz. In recent years, new technics have been developed to deposit graphene over a substrate and graphene films that have allowed the design of antennas at frequencies below 10 GHz. For example, T. Leng et al. [23] presented a dual-band graphene antenna for WiFi frequencies; this antenna was printed on a paper substrate. Furthermore, in [24] and [25], the authors showed graphene array antennas printed on paper for WiFi bands and Sub-6 GHz 5G mid-bands, respectively. Another example of printed graphene antennas was reported by K. Pan et al. [26], who designed an ultra-wideband monopole with printed graphene with a frequency range from 4 to 14 GHz.

Moreover, Lamminen et al. [27] shows a printed dipole antenna in Dupon dielectric and graphene flakes for ISM bands. An important application of graphene antennas is wearable technology [28,29,30]; Zu et al. [28] presented a design over a flexible substrate and highly conductive graphene film, and Sindhu et al. [29] employed laser-induced graphene (LIG) to print the design; both antennas operate at 5.8 GHz. Kapetanakis et al. [30] presented wearable patch antennas with graphene over a textile substrate at 2.45 GHz. 

Furthermore, taking advantage of the flexibility of graphene, Zelong et al. [31] reported a dual-band flexible antenna using a graphene film over a polydimethylsiloxane substrate. Finally, it is essential to mention that designs of graphene antennas over glass as a substrate barely exist. However, an example of graphene antennas over glass is reported in [32]. This paper presents a dipole-printed antenna over glass at 2.45 GHz. Table 1 summarizes the related work and the proposed antenna concerning operation frequencies, conductivity, type of graphene, and substrate.

This paper presents the design of a multiband coplanar waveguide antenna using a graphene film over a glass substrate. The proposed antenna presents omnidirectional radiation and operates in two bands, first in the 2.45 GHz ISM band and the second band covering the frequency range from 4 to 6 GHz to generate the multiband behavior; the antenna is formed by two resonator patches, as reported in [33,34,35,36]. The antenna is fully characterized by the reflection coefficient and the radiation patterns’ measurement. The simulated and measured results are in concordance. The main contribution of the proposal is the development of a multiband antenna over glass and graphene for integration into smart cities and vehicular network applications.

## 2. Materials and Methods

**Figure 1:**
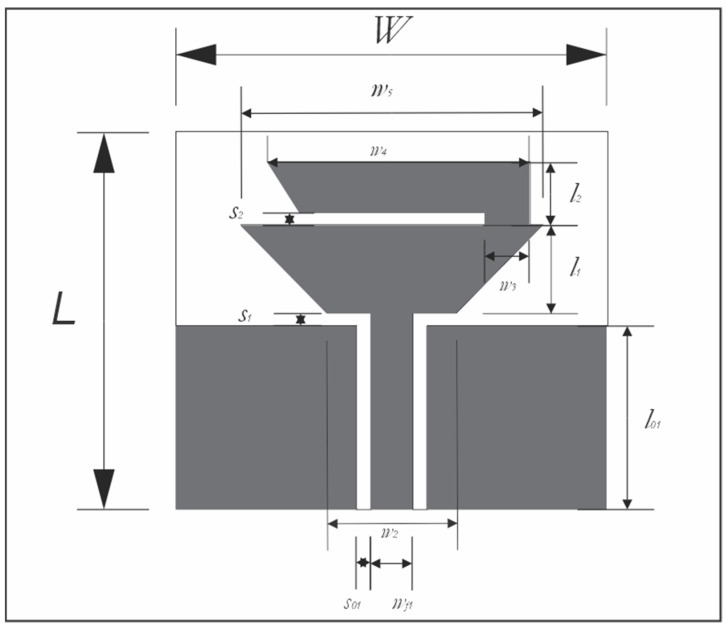
The geometry of the proposed antenna.

The proposed antenna is configured by a coplanar waveguide line to feed the antenna with a characteristic impedance *Z*_0_. In addition, the antenna has two radiator patches with an isosceles trapezoidal form [30,31,32,33]; Figure 1 shows the geometry of the proposed antenna. In this geometry, *w*_4_ controls the lowest frequency band (*f_min_*), and *w*_2_ works over the higher frequency band (*f_min_*). The dimensions of *w*_2_ and *w*_4_ were calculated by the following equations

(1) $$w_{2\;}=\frac{c}{2f_{max}\sqrt{\varepsilon_{r}}},$$

(2) $$w_{4\;}=\frac{c}{2f_{min}\sqrt{\varepsilon_{r}}},$$

where $\varepsilon_{r}$ is the relative permittivity of the substrate, and *c* is the speed of light in the vacuum.

In this proposal, for the antenna design, graphene foil was employed (XG Leaf B, Lansing, MI, USA) with a thickness of 240 μm and electric conductivity of 3.5 × 10^5^ S/m. The dielectric was glass with relative permittivity of 6 and a thickness of 2 mm. In order to obtain the design and optimize it, high-frequency structural simulation (Ansys HFSS, Canonsburg, PA, USA) was employed.

**Figure 2:**
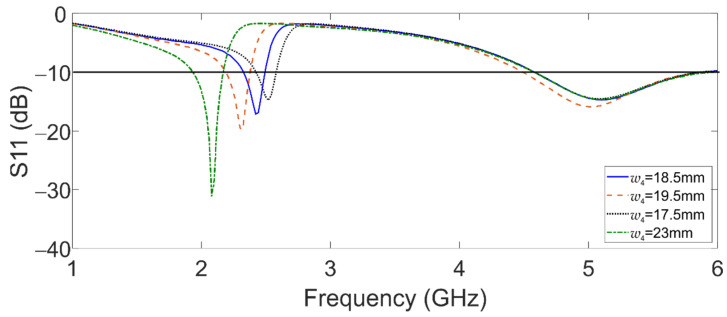
Simulated reflection coefficient of the dual-band graphene antenna for *w*_4_ variations.

The dimensions of the coplanar waveguide feed line were calculated using the Advanced Design System software (ADS, Santa Rosa, CA, USA), considering a characteristic impedance of 50 Ohms, and the dimensions of the trapezoidal patches were obtained with Equations (1) and (2). Considering the central frequency at 2.45 GHz for the first band and the second band at 5.8 GHz, the starting dimensions of *w*_2_ and *w*_4_ were approximately 10 mm and 25 mm, respectively. Throughout the parametric analysis of the reflection coefficient (S11), the final dimensions of the antenna were determined; these dimensions are reported in Table 2. The parametric analysis was developed by the variation in the parameters *w*_2_, *w*_4_, and *l*_01_. From this analysis, we found and demonstrated that *w*_4_ controls the band of 2.45 GHz; this resonance band shifts to the right as *w*_4_ decreases, as seen in Figure 2.

**Figure 3:**
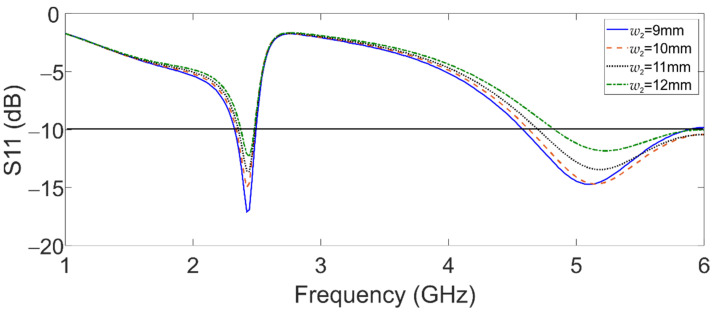
Simulated reflection coefficient of the dual-band graphene antenna for *w*_2_ variations.

**Figure 4:**
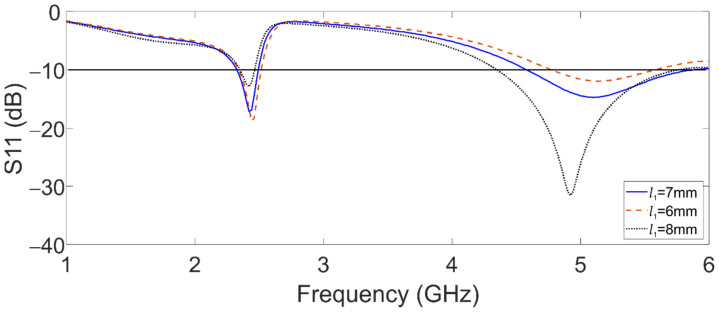
Simulated reflection coefficient of the dual-band graphene antenna for *l*_1_ variations.

Moreover, Figure 3 shows the effect of *w*_2_ over the upper band (5.8 GHz). Concerning this case, while *w*_2_ is reduced, S11 decreases, and the lowest frequency of this band shifts to the left. Finally, *l*_01_ affects both bands concerning the coupling, as shown in Figure 4.

## 3. Results and Discussion

After the final dimensions of the antenna were obtained through parametric analysis, the antenna was fabricated and fully characterized by the measurement of its reflection coefficient, radiation diagrams, and peak gain at 2.45 GHz and 5.8 GHz. 

**Figure 5:**
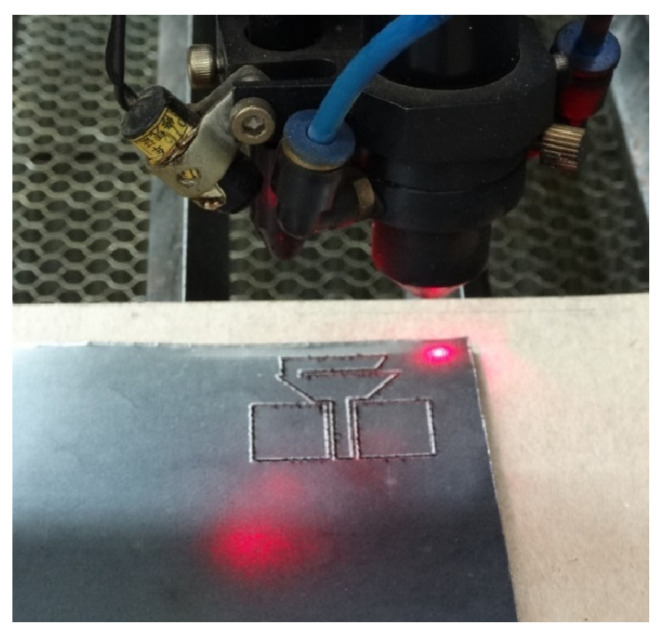
The process of recording the geometry of the antenna on the graphene film.

**Figure 6:**
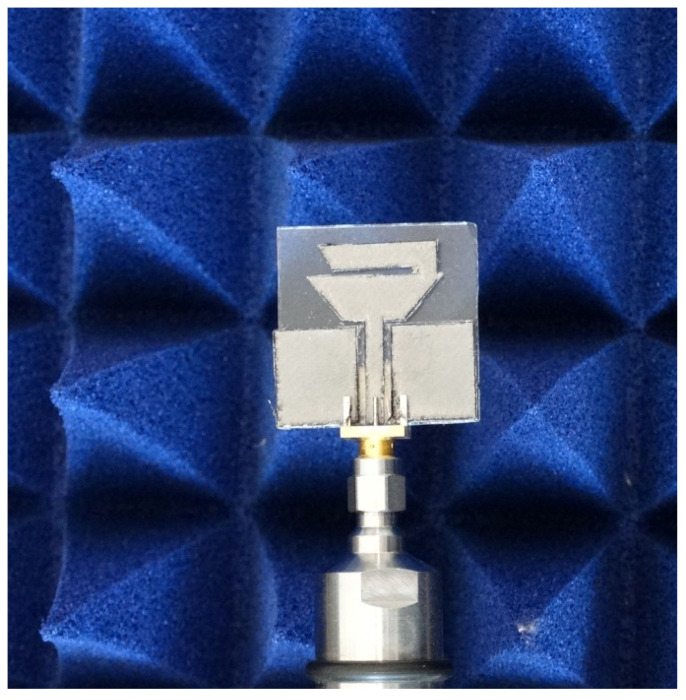
Fabricated dual-band graphene antenna.

The first step of fabricating the antenna was to record a mask of the desired geometry over the graphene foil using a laser cutter (SMTRobotic, 1390BT, China), as shown in Figure 5. Then with a double sided adhesive paper, the graphene mask of the antenna was placed over the glass substrate. In addition, to connect the antenna, an SMA connector was employed. Figure 6 shows the fabricated antenna.

**Figure 7:**
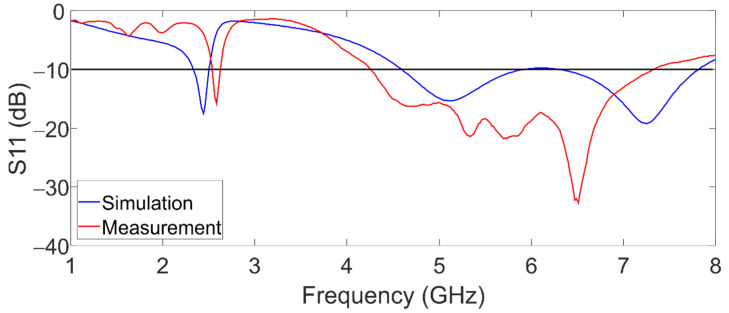
Simulated and measured reflection coefficient of the dual-band graphene antenna.

In other to start the characterization of the antenna, the antenna was connected to a Vector Network Analyzer (VectorStart, Anritsu, Morgan Hill, CA, USA) to measure its reflection coefficient (S11) from 1 to 8 GHz. The simulated and measured reflection coefficients are compared and shown in Figure 7. The measurement S11 suffers a shift of 150 MHZ for the first band, and the resonance frequency is at 2.6 GHz. The second band in the measurement starts at 4.3 GHz and ends at 7.1 GHz. These frequency shifts and differences between simulation and measurement are due to the variations in the dielectric properties of the glass substrate, and in a lower proportion to fabrication flaws. However, the bandwidth increase in the second band relates to the conductivity of the graphene in antennas with radiation elements with low conductivity such as ITO [16,17], present, and UWB behavior. The simulation and measurement are in concordance with the behavior of S11. Table 3 shows the fraction bandwidth for each band.

**Figure 8:**
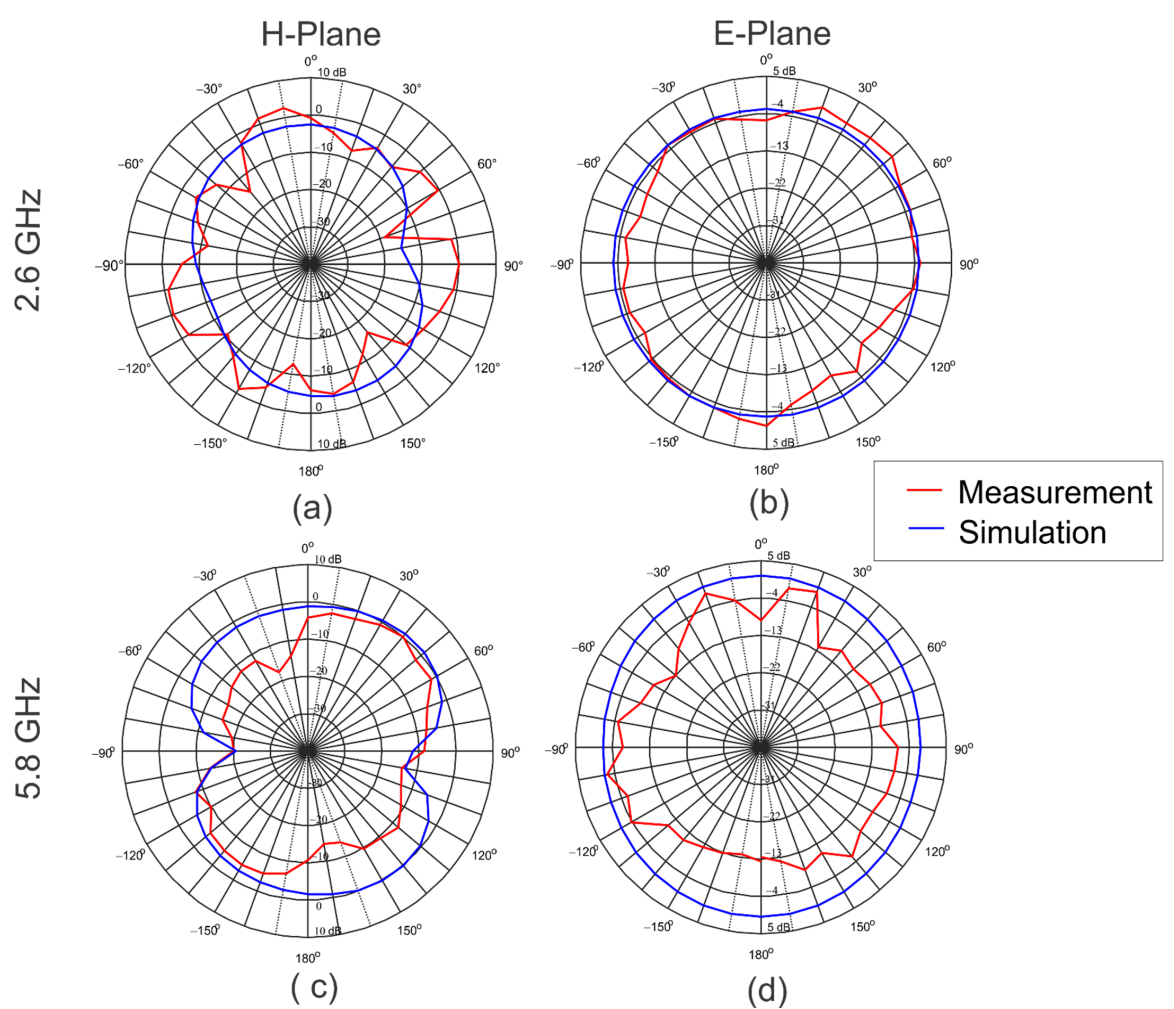
Simulated and measured radiation patterns: (**a**) H-plane 2.6 GHz, (**b**) E-plane 2.6 GHz, (**c**) H-plane 5.8 GHz, and (**d**) E-plane 5.8 GHz.

Figure 8 shows the measured and simulated radiation patterns in H and E planes for 2.6 GHz and 5.8 GHz. In both cases, the graphene antenna presents an omnidirectional behavior in the H-plane and bidirectional radiation in the E-plane. The resolution in degrees of the measurement of radiation patterns was 10 degrees. The difference between the simulated and measured radiation patterns is related to the uncertainty caused by the fact that the measurements were not performed in a controlled environment.

Finally, Table 4 shows the simulation and measured peak gain at two frequency bands. From this Table, we appreciate that the gain is negative at the frequency of 2.45 GHz; on the other hand, at 5.8 GHz, the gain increased to positive values. The gain was measured using the three antenna method [36]. The antennas employed in this gain measurement were the fabricated antenna, a standard log periodic directional antenna (Hyper Log 7060, Aaronia, Strickscheid, Germany), and a Vivaldi antipodal antenna that operates in the UWB range. These values of gain are expected; a similar behavior was reported in [25]. However, these values are higher than the gain of antennas with ITO [17].

**Figure 9:**
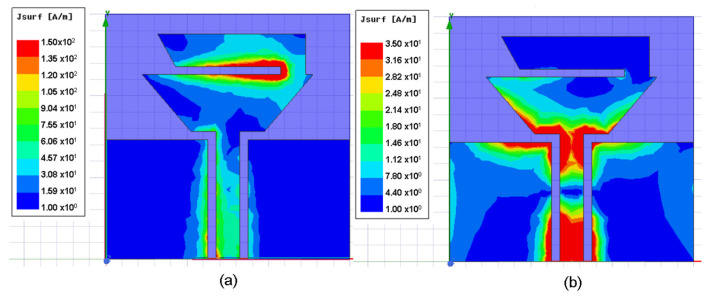
Simulated current distribution at (**a**) 2.45 GHz and (**b**) 5.8 GHz.

Finally, the current distribution for the graphene antenna was simulated at 2.45 GHz and 5.8 GHz and is shown in Figure 9. Figure 9a shows that the current distribution at 2.45 GHz is more intense in the second trapezoidal patch that corresponds to the lower frequency band. Moreover, at 5.8 GHz, the distribution current is mainly on the smallest base of the first trapezoidal patch; as was mentioned, this base controls the upper band of the antenna.

## 4. Conclusions

A dual-band CPW graphene antenna was designed for applications in smart cities and IoT. This antenna was designed using a graphene film and implemented over a glass substrate. The implementation of glass enables the proposed antenna to be integrated into applications of IoT and smart cities. This design consists of a coplanar line to feed the trapezoidal patches that produce the dual-band behavior. The measurement reflection coefficient is in concordance with simulations. 

The dual-band behavior of this antenna is presented in two bands. First, with regards to the lower band at 2.45 GHz, in the experimental results, this band suffers a smooth shift in frequency. The second band covers the ranges of 4 to 7 GHz. These two bands comply with several standards of communication.

Concerning the gain, these present values under 1 dB, in both frequencies, due to the conductivity of the graphene. Finally, the antenna presents an omnidirectional behavior for the H-plane.

## Figures and Tables

**Table 1:** Comparison between reporter works.

Reference	Frequencies	Graphene Type/Conductivity or Sheet Resistance	Dielectric
[23]	2.45 GHz/5.8 GHz	Flakes/3 Ω/sq	Paper
[24]	2.45 GHz/5.8 GHz	Flakes/3.5 × 10^4^ S/m	Paper
[25]	2.45 GHz/5.8 GHz	Flakes/3.5 × 10^4^ S/m	Paper
[26]	4 to 14 GHz	Flakes/7.13 × 10^4^ S/m	Paper
[27]	2.45 GHz/5.8 GHz	Flakes/4 Ω/sq	Kapton HN
[28]	5.8 GHz	Film/1.13 × 10^6^ S/m	Polydimethylsiloxane
[29]	5.8 GHz	LIG/7.18 × 10^2^ S/m	Polymide
[30]	2.45 GHz	Film/0.06 Ω/sq	Textile
[31]	2.45 GHz/5–7 GHz	Film/1.13 × 10^5^ S/m	Polydimethylsiloxane
[32]	2.45 GHz	Flakes/2.6 Ω/sq	Glass
This work	2.45 GHz/4–6 GHz	Film/3.7 × 10^5^ S/m	Glass

**Table 2:** Final dimensions of the proposed graphene antenna.

Parameter	Dimension (mm)	Parameter	Dimension (mm)
*L*	30	*l*_1_	7
*W*	30	*l*_2_	5
*w*_4_	17.5	*l*_01_	14.1
*w*_2_	9	*w*_5_	21
*w_f_*	2.91	*w*_3_	3.1
*S*_0_	1	*S*_1_	1
*S*_2_	1		

**Table 3:** Frequency ranges and fractional bandwidth of the dual-band graphene antenna.

	Frequency Range	Fractional Bandwidth
Simulated	2.45 GHz	4%
Measured	2.6 GHz	4%
Simulated	4.5 to 7.8 GHz	53%
Measured	4 to 7 GHz	54%

**Table 4:** Measured and simulated gain.

	Frequency (GHz)	Gain (dB)
Simulated	2.45	0.38
Measured	2.6	−0.2
Simulated	5.8	0.765
Measured	5.8	1
